# Influence of different processing techniques for prosthetic acrylic resins in the surface roughness parameters: a research article

**DOI:** 10.1186/s12903-024-04397-1

**Published:** 2024-05-30

**Authors:** Margarida Martins Quezada, Carlos Miguel da Costa Gomes Fernandes, Javier Montero Martín, André Ricardo Maia Correia, Patrícia Alexandra Barroso da Fonseca

**Affiliations:** 1 Faculty of Dental Medicine, Centre for Interdisciplinary Research in Health (CIIS), Estrada da Circunvalação, Universidade Católica Portuguesa, Estrada da Circunvalação, Viseu, 3504 – 505 Portugal; 2 Faculty of Engineering (FEUP), Universidade do Porto, Praça Gomes Teixeira, Porto, 4099-002 Portugal; 3 Faculty of Medicine, Department of Surgery, University of Salamanca, Patio de Escuelas, 1, Salamanca, 37008 Spain

**Keywords:** Polymers, Polymethyl methacrylate, Surface properties

## Abstract

**Background:**

Different processing techniques are employed to obtain poly (methyl methacrylate) (PMMA) with consistent surface quality in terms of topography and tribological function. The purpose of this research is to evaluate its influence on the surface height distribution.

**Methods:**

In this research, samples of conventional and CAD/CAM acrylic resins were prepared. The following surface roughness parameters were extracted from the profilometric readings: arithmetic mean roughness (Pa), skewness (Psk) and kurtosis (Pku). Profilometric profiles were additionally obtained.

**Results:**

The average roughness (Pa) with the conventional technique was significantly higher compared to CAD/CAM (t = 4.595; *P* < 0.001). Heat-cured resins presented the highest mean Pa (F = 6.975; *P* = 0.06). Heat-cured and milled resins show lower coefficient variation (CV) values, indicating more consistent surface finishing. The surface profiles revealed distinct characteristics in terms of skewness and kurtosis.

**Conclusions:**

The surface processing method, chemical composition and resin type significantly influence the surface finishing of the resin. The CAD/CAM resins exhibited superior results in terms of surface arithmetic mean roughness (Pa). However, heat-cured resin revealed to present the better surface consistency.

## Background

In accordance with Specification No. 12 of the American Dental Association (ADA) [1], polymers for prosthetic bases are classified into several types, depending on the polymerization reaction and their composition [2–5]. However, poly (methyl methacrylate) (PMMA) remains the most frequently used [4, 6, 7] due to its favourable characteristics, including processing and pigmentation, reduced toxicity and satisfactory mechanical properties [2].

Conventional PMMA is mainly accessible in the form of a powder-liquid system. The powder incorporates the polymer PMMA with the addition of additives, as pigments or acrylic synthetic fibers to mimic the aesthetics of oral tissues and to calibrate the physical properties [4, 6]. The liquid part contains a monomer of methyl methacrylate (MMA) in addition to cross-linking agents and inhibitors [4]. PMMA is derived from a polymerization reaction wherein the conversion of MMA into PMMA occurs during a curing process, activated either by chemical products, light or heat [5, 7].

Computer-aided design and computer-aided manufacturing (CAD/CAM) have been introduced as a method for producing PMMA for prosthetic bases [4]. A notable advantage appears to be the controlled temperature and pressure polymerization of prefabricated blanks of PMMA. Consequently, these materials are commonly referred to as high–performance polymers (HPPs) [3–5, 8].

Numerous researchers conducted comparisons of the properties between conventionally and CAD/CAM manufactured PMMA [3–6, 8]. The chemistry of CAD/CAM PMMA is similar to that of conventional heat cured PMMA [4, 9]. However, CAD/CAM PMMA exhibits advantages, including surface properties, flexural strength, and flexural modulus [3, 4, 6]. .

Different processing techniques are employed to obtain PMMA with the specified dimensional tolerances and surface quality consistency, to achieve the desired shapes [10, 11]. This must be examined from two perspectives: process control and tribological functionality [10, 12]. The functional properties are related to the 2D and 3D surface roughness, waviness and surface texture [13]. The surface topography is a random structure composed of microscopic peaks and valleys formed during the manufacturing process. As a result, macro roughness and micro roughness can occur [6, 11, 13].

The challenge arises to the necessity of selecting appropriate surface parameters to monitor whether the desired functional surface properties are achieved. The most common metric used to analyze surface roughness is Ra (arithmetical mean roughness). This parameter summarizes height variations; however, it lacks into surface shape and does not offer details regarding the frequency or regularity of occurrence [14].

Most surfaces exhibit a degree of randomness that may follow a Gaussian (normal) or non-Gaussian distribution. The specific characteristics of a surface’s height distribution are influenced by the method used to develop the surface. The Gaussian distribution has become a fundamental tool for classifying surface properties [15]. Surface parameters, such as skewness and kurtosis of the height distribution, are frequently used to characterize Gaussian topographies [16, 17]. Various authors have described the potential occurrence of identical Ra value for surfaces with different shapes and frequencies [15, 17].

This preliminary research intends to evaluate the influence of different processing techniques for prosthetic acrylic resins on the surface roughness parameters.

## Methods

Five denture base acrylic resins were selected as shown in Table 1. Five quadrangular-shaped specimens (20 × 20 × 3 mm) were manufactured according to the instructions and specific standards [18].

**Fig. 1:**
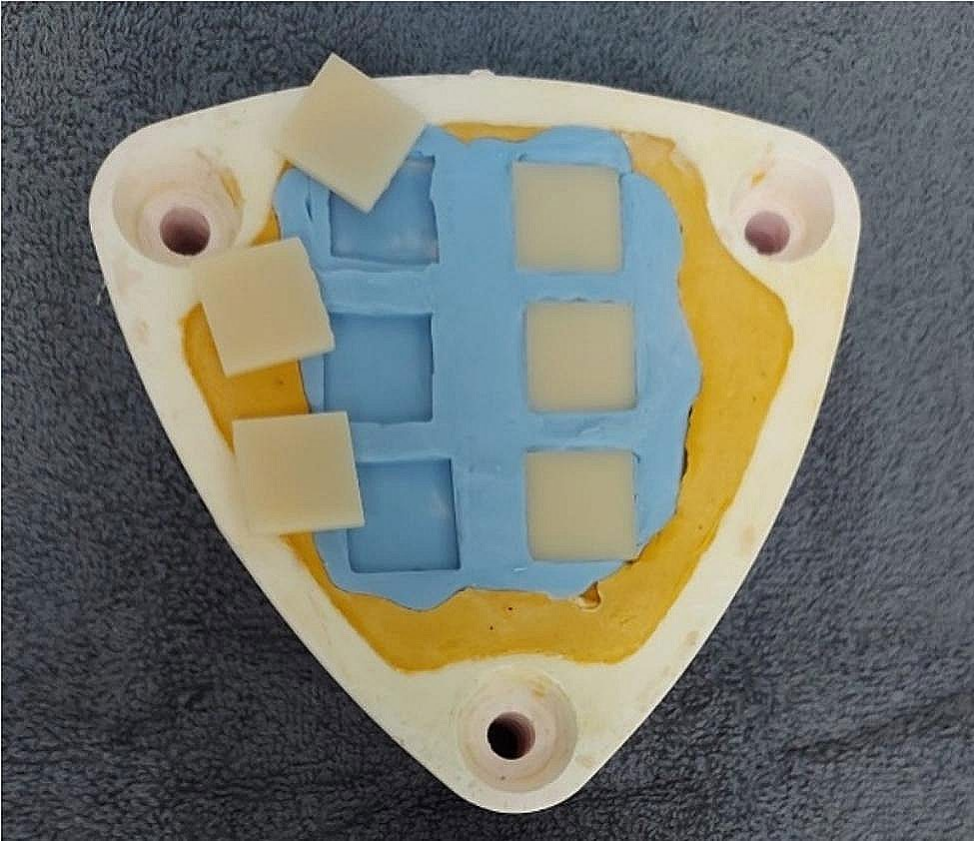
Silicone molds with predefined dimensions (20 × 20 × 3 mm)

For the specimens using conventional resin (self-cured, heat-cured and injected molded) (Table 2), silicone molds were prepared with the predefined dimensions (Fig. 1).

**Table 1:** Description of the resins used for this research

Name	Commercial brand	Country of origin
*Probase® cold*	Ivoclar Vivadent	Liechtenstein
*Probase® hot*	Ivoclar Vivadent	Liechtenstein
*iflex*™	tcs®	USA
CediTEC DB	VOCO® GmbH	Germany
V-Print dentbase	VOCO® GmbH	Germany

According to the manufacturer’s instructions, presented in Table 2, the self-cured denture base acrylic resin Probase*®* Cold and the heat-cured denture base acrylic resin Probase*®* Hot were obtained by a conventional flasking technique (Fig. 1). Both polymerization reactions were carried out in a pressure device for 30 min at 23 ºC and for 45 min at 100 ºC, respectively.

**Fig. 2:**
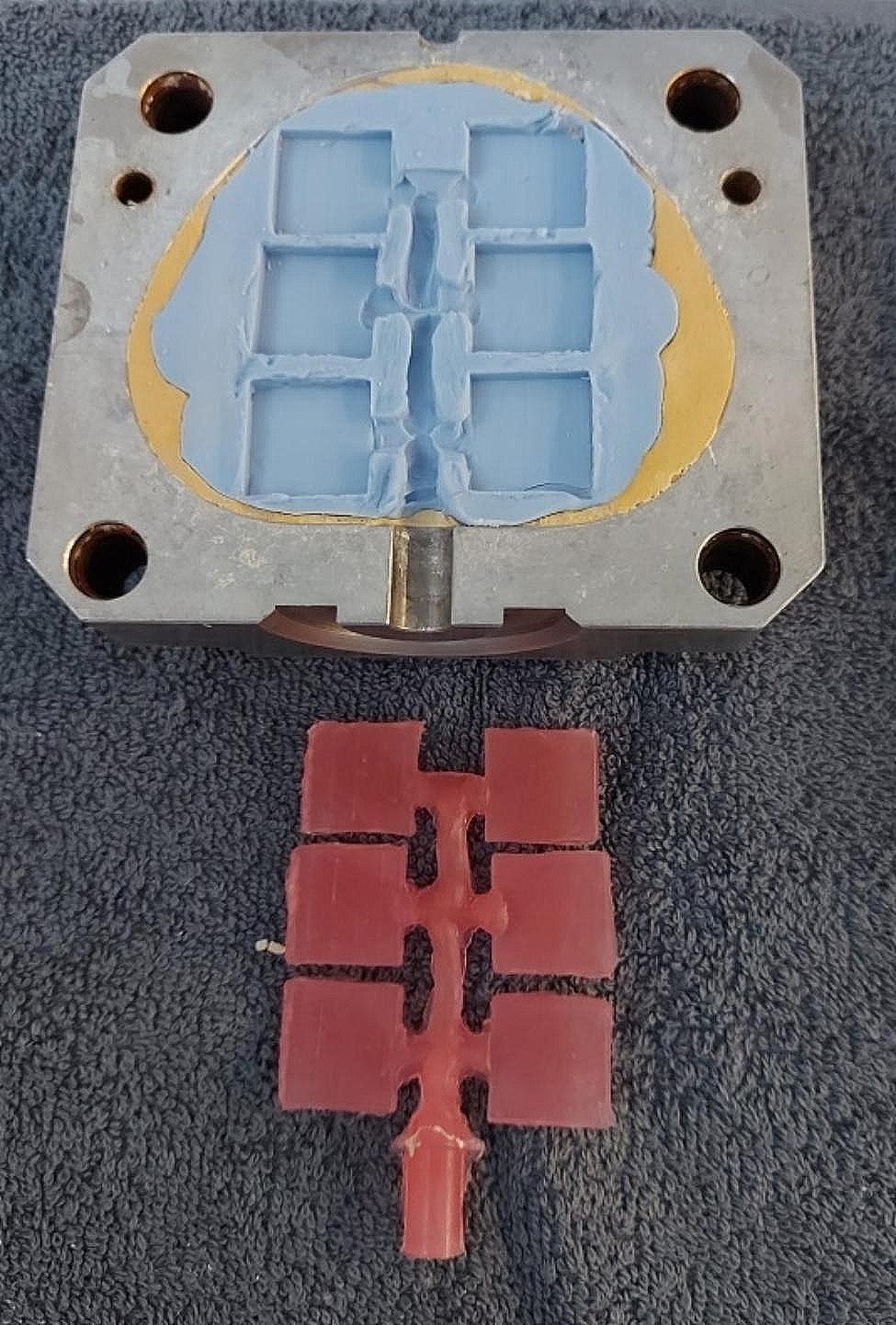
Injection technique on the predefined silicone molds

The resin tube iFlex™ was placed on tcs® Digital Furnace (tcs® Dental Inc., California) and injected with tcs® Handheld JP90 (tcs® Dental Inc., California) (Fig. 2). The polymerization occurred inside the muffle at 23 ± 2 ºC.

For the milled samples, the virtual design of the specimen was obtained with CAD software SolidWorks®, which was converted to a standard tessellation language (STL) file. Then, it was sent to a DWX-52D milling machine (DWX Series, Spain). A PPMA pre-polymerized block (*CediTEC DB*, VOCO®, GmbH, Germany) with 98, 5 × 30 mm dimensions was placed on the appropriate support for the size. A milling bur used at a 90º angle relative to the block position. The milling process was applied dry.

The 3D printed samples were virtually designed with CAD Asiga Composer (Asiga Composer, ASIGA, Germany), which was converted to an STL file. It was sent to an Asiga Max UV 3D printer (ASIGA, Germany). The specimens were obtained through the digital light processing method (DLP). After printing, the specimens were submitted to an ultrasonic bath with isopropyl alcohol for 2 min, and the post–processing procedure was executed with an Otoflash G171 flashing unit (NK-Optik GmbH, Germany): 10 flashes/second with a wavelength of 385 nm.

**Table 2:** Characteristics of the resins

Material	Composition		Ratio	Curing method
*Probase® Cold*	**P**: PMMA Dibenzoyl peroxide	**Li**: MMA	P/Li 20.5/10 (g/ml)	Self – curing: 23 ºC for 30 min (Conventional)
*Probase® Hot*	**P**: PMMA Dibenzoyl peroxide	**Li**: MMA 1–4 butanediol dimethacrylate	P/Li 22,5/10 (g/ml)	Heat – curing: 100 ºC for 45 min (Conventional)
*iFlex*™	Polyolefin			Injected molded (Conventional)
Ceditec DB	PMMA		99%	Milling (CAD/CAM)
V-Print dentbase	**Li**: UDMA Bis – EMA TEGDMA		50–100% 25–50% 5–10%	Digital light processing method (DLP) (CAD/CAM)

P – Powder; Li – Liquid; PMMA– Poly Methyl Methacrylate; UDMA– Urethane Dimethacrylate; Bis – EMA – Bisphenol-A-Ethoxylate Dimethacrylate; HEMA – Hydroxyethyl

No surface treatment was applied to any of the samples after processing, and sterilized compartments were used to avoid any interference or contamination. Then, the specimens were subjected to a profilometer (Hommelwerke LV-50 with linear unit and T800 controller, Hommelwerke, Germany) reading. The surface of the specimens was measured by a stylus probe with a diamond tip (length of 4.8 mm) at a constant speed of 0.5 m/s. The surface roughness parameters were directly obtained from the primary profile (Profile P). The use of Profile P is clinically relevant since it represents the curve formed when the actual surface of the material is cross-sectioned, without the use of a Gaussian filter [14].

The data were analysed using IBM^®^ SPSS^®^ Statistics for Macintosh, version 27 (IBM Corporation, USA). Pa roughness values are represented using the mean and standard deviation. A descriptive analysis was used to analyze skewness (Psk), and kurtosis (Pku). High kurtosis values indicate a sharp amplitude distribution with large peaks and valleys. A negative skewness suggests a concentration of the material near the top of the profile and a plateau-like surface. In addition, the percent variation coefficient (CV), defined as the ratio between the standard deviation and the average value was utilized. Two-way Analysis of Variance (ANOVA) with the Bonferroni post-hoc correction for a small sample size and predicted data not normally distributed was used to compare the distribution of surface arithmetic mean roughness (Pa) between different resin types. In order to test the means between two groups, Student’s t test was employed to assess differences in the distribution of Pa between pairs of resins.

## Results

Table 3 compares roughness data for different processing techniques and resin types. The overall mean roughness (Pa) for conventional techniques is 11.35 ± 4.68 μm, significantly higher than CAD/CAM techniques at 2.26 ± 1.29 μm (t = 4.595; *P* < 0.001), indicating that CAD/CAM yields lower overall Pa among resin types, heat-cured resins stand out with the highest mean Pa at 14.10 ± 4.80 μm, showing significant differences in inter-group comparison statistics (F = 6.975; *P* = 0.06). Post-hoc tests highlight significant differences, with heat-cured resins differing from 3D printed (*P* = 0.034) and milled resins (*P* = 0.015).

**Table 3:** Roughness data for different processing methods and resin types

Overall Pa (µm), mean ± SD							Inter-group comparison statistics
Conventional		CAD/CAM					
11.35 ± 4.68		2.26 ± 1.29					t = 4.595; *P* < 0.001
**Resin-specific Pa (**µ**m), mean** ± **SD**						**Inter-group comparison statistics**	
Self-cured	**Injected molded**	**Heat-cured**	**3D printed**		**Milled**		
11.77 ± 5.26	8.19 ± 3.20	14.10 ± 4.80ª.^b^		3.00 ± 1.48^b^	1.51 ± 0.56ª		F = 6.975; *P* = 0.06

a, b: significant differences in pairs of resins using Bonferroni post-hoc correction at *P* < 0.05

Table 4 provides an analysis to the surface roughness measurements for dental acrylic resins and the corresponding processing techniques. The variation coefficient (CV) indicates variability in roughness. 3D printed and self-cured resins have higher CV values (3D printed: CV = 0.493, self-cured: CV = 0.447), suggesting greater variability. In contrast, heat-cured (0.341) and milled resins show lower CV values (0.371). Surface profiles also reveal distinct characteristics. Self-cured (-0.453) and milled (-0.135) resins have a plateau-like surface with negative skewness, while injected molded resins show sharp variations with positive skewness (0.872) and substantial kurtosis (1.068).

**Table 4:** Measures of roughness dispersion for polishing techniques and resin types

Resin types and polishing techniques	Variation coefficient (CV)	Skewness (Psk)	Kurtosis (Pku)
Conventional			
Self-cured	0.447	-0.453	0.462
Injected molded	0.391	0.872	1.068
Heat-cured	0.341	0.339	0.267
CAD/CAM			
3D printed	0.493	0.201	1.578
Milled	0.371	-0.135	0.138

**Fig. 3:**
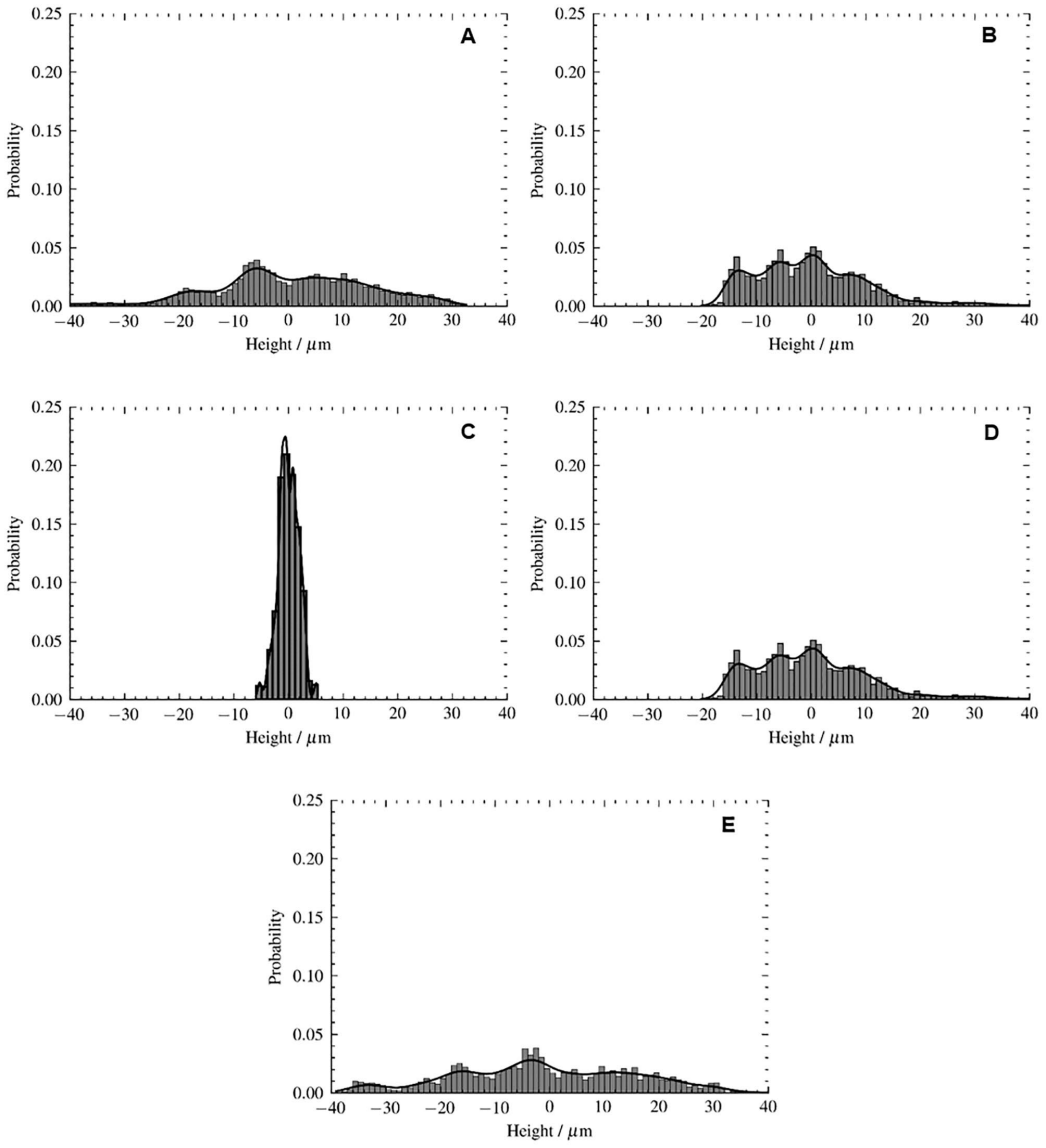
Probability of having a given height of the surface for the specimens according to resin processing type: **A** (self-cured), **B** (heat-cured), **C** (3D printed), **D** (milled), **E** (injected molded)

Surface profiles also reveal distinct characteristics (Fig. 3).

Self-cured (-0.453) and milled (-0.135) resins have a plateau-like surface with negative skewness, while injected molded resins show sharp variations with positive skewness (0.872) and substantial kurtosis (1.068) (Table 4).

## Discussion

In terms of analysis of the surface properties, the authors proposed a different approach to the assessment using the following parameters: skewness (Psk), kurtosis (Pku) and arithmetic mean roughness (Pa) [14]. The validation of the use of Pa instead of Ra, commonly used, had already been demonstrated [14, 19, 20]. The results published by the authors of the present research were reused for the present analysis [14].

Considering the existing drawbacks between the mechanical and physical properties of conventional PMMA, new processing methodologies were developed [4, 8, 9, 21]. Superior surface properties in comparison to conventional PMMA may be attributed to the unique processing method of the CAD/CAM PMMA in which high temperatures, pressure and lower levels of residual monomer are used to obtain pre-polymerized PMMA, in case of subtractive technique, or a layer by layer polymerization, in case of additive technique [5, 6, 21]. In general, when compared the two processes in relation to surface roughness, a significant difference in mean Pa (t = 4.595; *p* < 0.001) highlights the processing method impact on surface finishing. In the analysis in pairs, heat-cured differs significantly from 3D printed (*p* = 0.0034) and milled resins (*p* = 0.015).

Considering the chemical composition, several studies reveals that the composition of the CAD/CAM PMMA is similar to that of conventional PMMA [4, 9]. In contrast with what is presented in Table 2, also the chemical composition of PMMA polymers reveals influence on the surface roughness. Heat cured resins reveals the highest values of Pa in contrast with the results obtained by Berger et al. [22]. . The reaction of polymerization is activated by heat, therefore it is expected that the degree of conversion of MMA monomer occurs almost totally. Additionally, the polymer that constitute has a lower granule size in comparison with self cured resins, for example. A direct effect on the surface roughness by the reaction initiatior can be established as this resins uses benzoyl peroxide and 1–4 butanediol dimethacrylate [6, 22].

When assessing the overall roughness level, surface height distribution symmetry is a crucial aspect related to surface characteristics. As a result, it has the potential to measure the consistency of surface texture [16, 23–25]. Probability density and distribution curves are determined upon the nature of the processing method [15]. The variation coefficient is a measure of dispersion in relation to mean values in this study related to the surface roughness (Pa). The results of CV reveal lower variation on the overall Pa in milled (CV = 0.371), heat-cured resins (CV = 0.341) which means a higher probability density and a lower dispersion of Pa values in the distribution curve. The opposite appears in the self cured resins (CV = 0.447) and 3D printed (CV = 0.899). In terms of texture considerations, a lower CV indicates a more homogeneous surface. Heat-cured resin presented the higher Pa values with the lowest CV, indicating a more consistent surface quality, in comparison with milled resin with the lowest values of Pa. The opposite also occurs for hight CV value with a more heterogenous surface.

The correlation skewness (Psk) and kurtosis (Pku) provides valuable insight for analyzing the symmetry of a texture amplitude and to understand whether it contains inordinate high peaks/valleys on the surface and its influence on the bacterial adhesion [10, 15, 26, 27]. A non–Gaussian distribution of the roughness profile is characterized by Psk and is responsive to sporadic deep valleys or high peaks, as it quantifies the symmetry of the profile distribution with respect to its central line [10, 15]. Negative skewness pertains to profiles that are more prevalent in deep valleys, as occurs in self-cured (Psk = -0.453) and milled (Psk = -0.135). Further, non-Gaussian surfaces with relatively flat peaks and valleys are indicated by a Pku value less than 3, presented in all types of resins. Injected molded resins contrast with sharp variations with positive skewness (0.872) and substantial kurtosis (1.068). The higher Pku value indicates that the surface contains extreme peaks or valleys [23].

Despite the fact that the present research presents an “in vitro” design, further studies should proceed with the evaluation of the clinical implication related to the parameters Pa, Psk and Pku. First, in terms of selecting the appropriate polishing protocol for each type of acrylic resin, considering different processing techniques, their chemical composition, and consequently, the implications in terms of surface properties, such as the behaviour of roughness along the profile of a surface. Second, there is a lack of consensus regarding the minimum level for microbial adhesion and could differ according to the acrylic used and the hability of the microorganism to adhere to different surfaces [26, 27]. Studies report that microorganisms appear to have a preference for adhesion on surfaces with scratches and grooves and not necessarily with higher Pa values [26]. Therefore, the surface topography may have a greater influence on the bacterial adhesion than the roughness parameter value itself. A consensus should be establish on microbial adhesion thresholds and explore the interaction between surface topography and bacterial adherence.Two main limitation can be displayed. First, only five commercial brands of acrylic resins for prosthetic bases were tested in terms of composition and processing technique. Second, in relation to the shape of the test specimens, the quadrangular-shaped specimens do not resemble the complexity shape of the prosthetic bases at the clinical level.

## Conclusions

This research undertook a comprehensive analysis of surface properties in acrylic resins using the parameters Pa, skewness and kurtosis. The surface processing method has a direct influence on the surface behavior. The distribution height curve characterizes the surface topography of manufacturing procedures. CAD/CAM resins exhibited superior results in terms of surface roughness, although, heat-cured resin revealed to present a better surface consistency. A focus on achieving optimal surface properties should extend to the selection of appropriate polishing protocols based on resin’s type, processing technique and chemical compostion.

## Data Availability

All data generated or analysed during this study are included in this published article.
